# The behavior of Broad-tailed hummingbirds is altered by cycles of human activity in a forested area converted into agricultural land

**DOI:** 10.7717/peerj.14953

**Published:** 2023-02-28

**Authors:** Verónica Mendiola-Islas, Carlos Lara, Pablo Corcuera, Pedro Luis Valverde

**Affiliations:** 1 Universidad Autónoma Metropolitana, Iztapalapa, Doctorado en Ciencias Biológicas y de la Salud, Ciudad de México, México; 2 Centro de Investigación en Ciencias Biológicas, Universidad Autónoma de Tlaxcala, San Felipe Ixtacuixtla, Tlaxcala, Mexico; 3 Departamento de Biología, Universidad Autónoma Metropolitana-Iztapalapa, Ciudad de México, México

**Keywords:** Disturbance, Hummingbirds, Territorial behavior, Urban ecology, Weekend effect

## Abstract

**Background:**

By changing the circumstances in which animals make their behavioral decisions, weekly cycles of human activity might cause changes in wildlife behavior. For example, when there is more human activity in a location, animals may become more vigilant, which can decrease the time they spend foraging, or roam farther from home, leading to increased home range size. Overall, there has been little exploration of how animal species living in locations that have undergone land use change are affected by the temporal dynamics of human activity levels. In this study, we aimed to analyze the effect of the weekend on agricultural activities and hummingbird territorial activity. We examined differences between weekdays and weekends in factors previously shown to follow weekly cyclical patterns, such as pedestrian presence, traffic, and the presence of domestic animals. We hypothesized that territorial hummingbirds would respond to these weekly cycles of human activity by altering their behavior.

**Methods:**

We studied Broad-tailed hummingbird territories in forested areas that had been transformed to agriculture lands in central Mexico. We evaluated whether territorial individuals changed their behaviors (*i.e.*, chases of intruders, foraging within their territory, number of intruders allowed to forage in the territory) in response to variation between weekdays and weekends in the number of pedestrians, cyclists, dogs, farm animals and vehicles.

**Results:**

We found that the level of agriculture-related human activities showed a weekly cycle at our study site. On weekdays there was higher traffic of pedestrians, cyclists, dogs, farm animals and vehicles, compared to the weekends. Hummingbirds responded to these weekday-weekends differences by changing their territorial behavior. Compared to weekends, on weekdays hummingbirds showed a decrease in defense (number of chases) as well as the use of their territory (number of flowers visited), which allowed increased access to intruders (number of visited flowers by intruders).

**Conclusions:**

Our findings suggest that variation in agriculture-related human activities between weekdays and weekends can alter the territorial behavior of hummingbirds. Behavioral shifts seem to be related to these human activity cycles, leading hummingbirds to reduce chases and feeding during weekdays when human activity is highest, but increasing both behaviors during times of minimal disturbance.

## Introduction

Human activity generally follows some degree of scheduling, which is essentially a socially constructed artifact determined by arbitrary social conventions; *e.g.*, shops open at 8 am, markets close at 9 pm, or Monday through Friday are work days, and Saturday and Sunday are rest days (Zerubavel, 1985). This routine in people’s lives leads to variations in vehicular traffic, noise, and pedestrians in particular places, and it is possible to imagine that animals whose habitats overlap with human presence may respond flexibly to such cues. In other words, cycles of human activity may influence animal behavior patterns. This idea is called the “weekend effect hypothesis”, named after the Stalmaster & Kaiser (1998) study. In the Skagit River Bald Eagle Natural Area (SRBENA) in northwest Washington, USA, these authors investigated how outdoor activities affected bald eagles (*Haliaeetus leucocephalus*) that were wintering there. Their results showed that recreational activity reduced eagle numbers in the SRBENA, and these reductions were greatest on weekends or other days of intense human activity. Likewise, their finding that eagles’ use of the river decreased on weekends and then increased after the weekend strongly suggested that eagles were displaced from the river by recreationists and therefore lost access to food.

Indeed, the “weekend effect”, which occurs when more people visit a recreational location on weekends and during holidays and create more disturbance to the local wildlife, can affect animals that live in parks and preserves. The more people there are on specific days, the more likely it is that animal behavior will shift. Animals often avoid human-visited areas which means they become more vigilant (Tarjuelo et al., 2015), which can decrease the time they spend foraging (Payne et al., 2014), or cause them to roam farther, which may increase their home range size (Perona, Urios & López-López, 2019). Overall, this dynamic level of human activity has been shown to affect animals’ behaviors in natural environments (Blumstein, 2014), but there has been little exploration of how animal species living in urban habitats or those locations that have undergone land use change are affected by the temporal dynamics of human activity levels (*e.g.*, Fernández-Juricic & Tellería, 2000). For example, Bautista et al. (2004) found that Spanish Imperial Eagles (*Aquila adalberti*) and vultures changed their behaviors during weekends due to an increase in car traffic in southwestern Madrid Province of central Spain, but other raptors did not. Similar differences were found across a wide range of bird species, although not uniformly within each genus (*e.g.*, Donázar, Ceballos & Cortés-Avizanda, 2018; Perona, Urios & López-López, 2019). Similarly, a study of bats in an urban area from Greensboro, North Carolina, USA (Li et al., 2020) found changes in activities such as flying movements and roost that appear to be linked to weekly cycles of human activity. In contrast, other studies have shown no apparent change in animal behavior based on weekly human activity cycles, such as the Diniz, Silva-Jr & Macedo (2021) study on urban Rufous Hornero (*Furnarius rufus*) duets in Brazil. The lack of significant effects could mean that these species have adapted to the dynamic changes brought on by human activity cycles, or it could mean that there is not enough variance in human activity levels between the weekdays and weekends to cause behavioral changes.

In agricultural activities, preparation of farmland and crop cultivation generally entail activities during the weekdays, which although they are not completely stopped, can be strongly reduced during the weekends (Jean et al., 1988). This variation in the intensity of working activities throughout the week also means that the presence of people, farm animals, dogs, and vehicles (*e.g.*, cars, tractors and other agricultural machinery) also fluctuates. Many agricultural lands are ideal habitat for a large number of herbaceous and disturbance-tolerant plant species, and several of these plants have flowers that are highly attractive to hummingbirds, which can establish foraging territories and defend them against intruders over several days or weeks (Lyon, 1973; Lara, 2006; Leimberger et al., 2022). A territorial hummingbird should confront the intruders mainly through chases to lead them away from their territory and decrease the likelihood of the intruder returning (Camfield, 2006). The chase may be energetically costly and leaves the territory owner unable to defend against other intruders during the chase, however, this behavior is commonly used because it is an effective deterrent and reduces the risk of there being a fight (Wolf & Hainsworth, 1971). In this context, our study aimed to investigate the weekend effect on agricultural activities and hummingbird territorial activity. We examined variation between weekdays and weekends in factors that have previously been shown to follow a weekly cyclical pattern, including pedestrian presence (Fernández-Juricic & Tellería, 2000), traffic flow (Williams, Durvasula & Brown, 1998; Gross, Pasinelli & Kunc, 2010) and the presence of domestic animals (*e.g.*, dogs and farm animals; Weston et al., 2014). Here, we hypothesized that territorial behaviors in hummingbird males (*i.e.,* chases of intruders, foraging on their own territories, and the number of intruders allowed to forage in the territory) should be different in response to weekday-weekend variation in human activity levels. We predicted that territorial owners would decrease their foraging and defensive behaviors on days with higher human activity levels (*i.e.,* weekdays), compared to days with lower human activity (*i.e.,* weekends).

We studied individual hummingbird territories within the limits of La Malinche National Park (hereafter referred as LMNP), Tlaxcala, Mexico. Even though it is a protected natural area, the edges of LMNP face strong anthropogenic pressure, leading to the historic transformation of oak and pine forest to agricultural activities, mainly maize cultivation and grazing of cows, sheep and goats. This disturbance has promoted a landscape with cultivated lands, generally surrounded by areas of secondary vegetation, where the hummingbird-pollinated Firecracker bush (*Bouvardia ternifolia*), is abundant throughout the summer. We tested our hypothesis in male Broad-tailed hummingbirds (*Selasphorus platycercus*). This species is a long-distance migrant, but there are resident populations in Central Mexico, where it is relatively abundant in summer (Hernández-Hernández et al., 2022).

## Materials & Methods

### Study area

**Figure 1:**
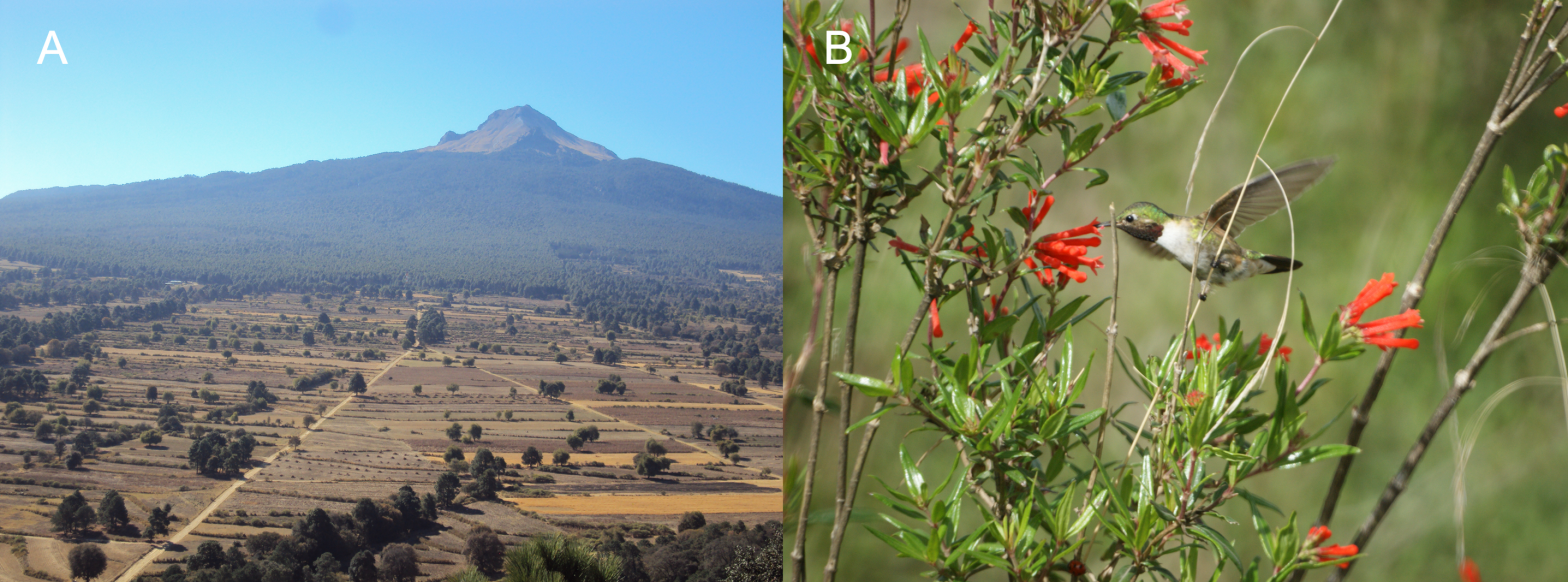
The type of vegetation where the territories of the Broad-tailed hummingbirds were monitored. (A) The study was carried out in areas where land use was changed from pine-oak forest to rainfed agriculture and induced pasture, traversed by an extensive network of roads and trails that vary in intensity of human, animal (livestock, dogs) and mechanical (vehicles, tractors) traffic. (B) These roads form a mosaic of flowering patches of different sizes of the shrub *Bouvardia ternifolia*, one of the main floral resources for this hummingbird species. Photo credit: Carlos Lara.

Hummingbird territories were studied from June to August 2016, in an area at the border of LMNP, Tlaxcala, Mexico. LMNP forms part of the Transmexican Volcanic Belt. It has an area of 46,096 ha, located between 19°14′N and 98°14′W, with an altitude ranging from 2300 to 4461 m above sea level (m.a.s.l.) (López-Domínguez & Acosta, 2005). The study area, which is between 2,200 and 2,300 m.a.s.l., has a transitional vegetation type from protected oak forest to rainfed farmland and induced pasture. The oak trees (*Quercus laurina*, *Q. crassifolia*, and *Q. rugosa*), which dominate the forest, grow in stands with 60 to 70% coverage. A mosaic of induced pasture and secondary vegetation from the original burned forest or abandoned farmed areas makes the area of land use shift from oak forest to grassland (Fig. 1A). The most common species are *Festuca tolucensis*, *Muhlenbergia macroura* and *Stipa ichu* (Lara, 2006; Martínez-García, Lara & Ornelas, 2013).

LMNP is accessible from urbanized areas by an extensive network of roads and trails, which vary in the intensity of human, animal (livestock, dogs) and mechanical (vehicles, tractors) traffic. Within the limits of the park, these roads form a mosaic of patches of flowering vegetation with different sizes of Firecracker bush, one of the most important ornithophilic plant species in this area during the summer months, which is associated with secondary vegetation (Lara, 2006). The Broad-tailed hummingbird is a small hummingbird, measuring 8–9 cm in length and weighing 3–4 g (Arizmendi & Berlanga, 2014). It inhabits open areas with shrubs and bushes in pine, pine-oak and juniper forests (Arizmendi & Berlanga, 2014). In the study area, it establishes feeding territories in floral patches of *B. ternifolia* (Fig. 1B), which it defends against conspecifics as well as other hummingbird species. This includes two resident species—the White-eared hummingbird (*Basilinna leucotis*, length: 9–10 cm and weight: 3.2–3.6 g) and the Magnificent hummingbird (*Eugenes fulgens*, 11–14 cm and 7–8 g)—and three migrant species—the Green violet-ear hummingbird (*Colibri thalassinus*, 10.5–11.5 cm and 4.8–5.7 g), the Blue-throated hummingbird (*Lampornis clemenciae*, 11–12 cm and 6.8 g) and the Rufous hummingbird (*Selasphorus rufus*, 7–9 cm and 2–5 g) (Lara, 2006).

The field research reported here was carried out with the necessary permits issued by the Mexican government (SEMARNAT No. FAUT-0296).

### Territorial and foraging behaviors

We used the following criteria to determine that a floral patch of *B. ternifolia* was an actively defended feeding territory: (1) the territory owner always returned to the same perch near the patch, (2) foraged within the patch, and (3) actively defended the patch through chases (Camfield, 2006; Mendiola-Islas et al., 2016; Márquez-Luna et al., 2019; Márquez-Luna et al., 2022). A chase implies persecutions and aggressions towards an intruder to try to force it away from the territory. In our field observations, we did not tag hummingbirds because the tiny size of the permanent bands commonly used to tag hummingbirds makes it impossible to identify them individually (Márquez-Luna et al., 2022). Instead, during the behavioral recording, each territory owner was recognized based on the fact that chases started from a certain perch and that the same perch was frequented. Twenty-one territories of adult male Broad-tailed hummingbird were monitored in an area of about 50 hectares.

In each territory, the territory owner’s behavior was observed and recorded for a period of four continuous hours (from 8:00 to 12:00 h), when hummingbirds are more active foraging and nectar production is high in this plant species (Torres et al., 2008). Each territory had the same sampling effort on weekdays and weekends from June to August. Due to the size of the territories (see below), the observations were carried out using binoculars (10 × 42), standing from different points 10 m away from each territory. We detected no apparent approach or avoidance behaviors by birds in response to the observers. We recorded: (1) the number of times the territory owners chased an intruder, and (2) the number of intruders that were not chased and were able to forage in the territory. Additionally, (3) we recorded the number of flowers visited by the owners inside their territories during the entire observation period.

### Size and quality of the territory

To estimate the size of the territories (area), we observed the behavior of the owner hummingbird and the locations of the perch, foraging, and chases. Once the observation period was over, we used a GPS (Garmin) to mark points around the perimeter of each activity performed by the territory owner, then calculated the area (square meters) encompassed within the perimeter points. During the observation period, we also counted the number of open flowers contained in each monitored territory.

### Human activities

In the study site, agricultural activities are carried out from June to August, which include preparing the land for plowing and cultivation of maize, as well as using non-arable land for grazing. Farmers are usually accompanied by domestic dogs, and the main modes of transportation are walking, vehicles (*e.g.*, tractors or cars), and bicycles. These activities had an apparent pattern throughout the week, with high activity on weekdays and low activity on weekends. Thus, we recorded the levels of human activity in the study areas in order to support our classification into activity levels on weekends and weekdays (sensu Diniz, Silva-Jr & Macedo, 2021). At the same time as the behavior of hummingbirds in a territory was recorded, we quantified the levels of human activity by counting: (1) number of pedestrians plus cyclists, (2) number of dogs plus farm animals and (3) number of vehicles. Thus, this sampling was carried out over the same four continuous hours and on the same days that each territory was monitored. All these activities had to occur within or as close as ∼ 5 m from a territory to be considered.

### Statistical analyses

We used generalized linear mixed models (GLMMs, package glmmTMB, Brooks et al., 2017) using a Poisson error distribution and logarithmic link function in R 4.0.0 (R Core Team, 2020) to analyze our count data. First, we examined the impact of the days of the week (predictor) on the number of pedestrians and cyclists, then the number of dogs and farm animals, and finally the number of vehicles (response variables), in order to confirm our two levels of human activity classification (weekdays and weekends). Each model had the territory identity as a random effect.

After corroborating the difference between weekends and weekdays in the intensity of human activities, we analyzed the effect of these human activity levels on hummingbird territorial behaviors (response variables), measured as the number of chases (defensive activity), the number of flowers visited in the territory (foraging activity), and the number of intruding hummingbirds that foraged inside the territory (failed defensive activity). For each model, we included human activity level (weekday or weekend) as a fixed effect and the territory identity as a random effect. The species that territory owners chased were *Eugenes fulgens*, *Colibri thalassinus*, *Lampornis clemenciae*, *Basilinna leucotis* and *Selasphorus rufus*, which were all pooled for our analyses. Because the size of a territory and the number of flowers it contains can affect defensive behavior in hummingbirds (*e.g.*, Hixon, Carpenter & Paton, 1983; Camfield, 2006; Mendiola-Islas et al., 2016), these variables were added as covariates in the models.

We obtained standardized model coefficients (*β*) to evaluate the significance of predictor variables. Additionally, we calculated pseudo marginal *r*^2^ for each model on territorial behavior (Nakagawa & Schielzeth, 2013).

## Results

### Human activity levels differed between weekdays and weekends

**Figure 2:**
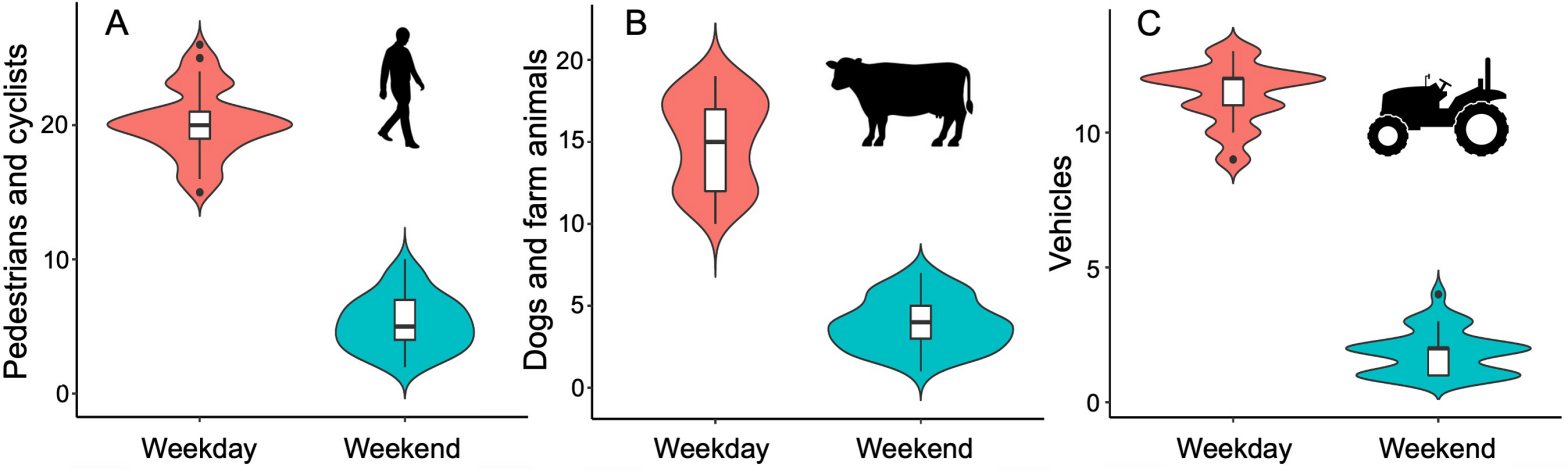
Variation of human activity levels recorded at the study site. Violin plots showing the kernel probability density of the data at different values to illustrate differences between weekends and weekdays in the number of pedestrians and cyclists (A), dogs and farm animals (B) and motor vehicles (C). The box plots within each violin plot denote the median and interquartile range along with outliers. At each hummingbird territory (*n* = 21 territories), these human activities were monitored for a period of four continuous hours (0800–1,200 h) on weekdays and weekends.

The number of pedestrians and cyclists (*β* ± se = −1.35 ± 0.06; *r*^2^ = 85.3%), number of dogs and farm animals (*β* ± se = −1.36 ± 0.07; *r*^2^ = 81.5%), and number of vehicles (*β* ± se = −1.82 ± 0.10; *r*^2^ = 84.7%) were higher on weekdays than on weekends (Fig. 2, Table 1). These results support variation in human activity levels over the course of the week at our study site.

**Table 1:** Results of generalized linear mixed models (GLMM) with Poisson error distribution to test for differences between weekends and weekdays on the human activities sampled in Broad-tailed hummingbird territories (*n*= 21) at the study site. Data for the numbers of (a) pedestrian and cyclists, (b) dogs and farm animals, and (c) vehicles were collected on nine surveys through the study. Significant results are in bold.

	Estimate	se	*z*-value	*Pr* (>|*z*|)
(a) Pedestrian and cyclist				
(Intercept)	0.81	0.52	1.56	0.116
Human activity level	1.65	0.80	20.60	**<0.001**
(b) Dogs and farm animals				
(Intercept)	2.70	0.30	8.90	**<0.001**
Human activity level	1.04	0.03	27.03	**<0.001**
(c) Vehicles				
(Intercept)	3.41	0.42	8.08	**<0.001**
Human activity level	−1.60	0.06	−25.39	**<0.001**

### Hummingbird territorial behavior is altered by human activity levels

**Figure 3:**
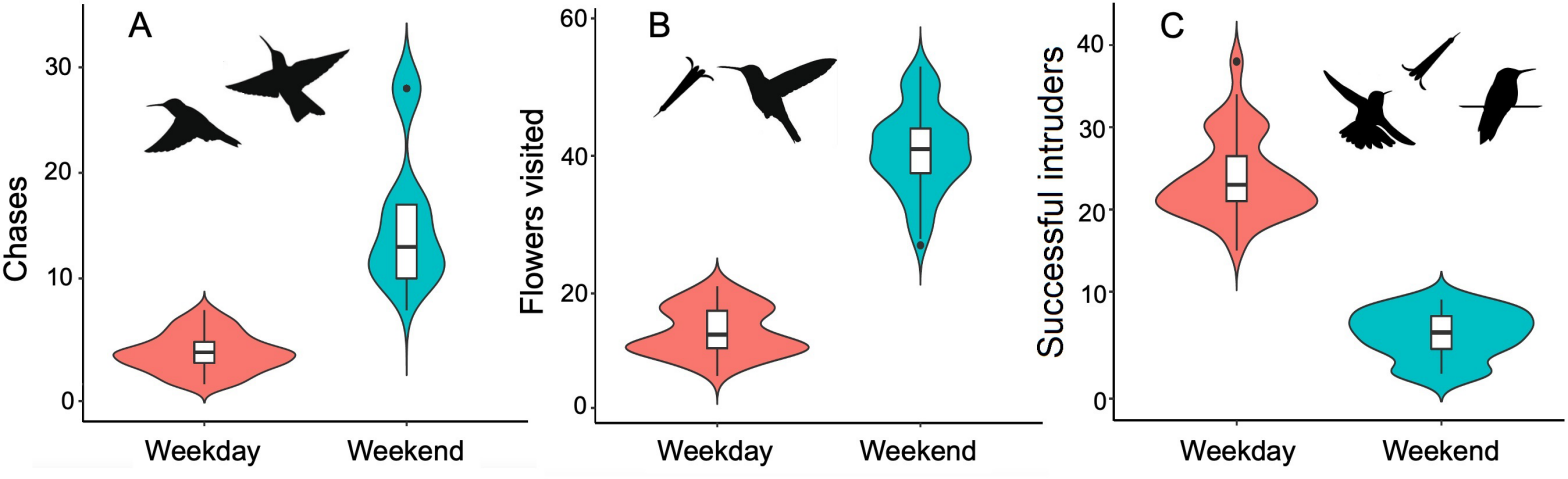
Changes in behavior recorded in territorial hummingbirds surveyed on weekdays and weekends. Violin plots showing variation in the number of chases of intruders (A), the number of flowers visited by the territory owner (B), and the number of intruders foraging in the territory without being chased by the owner (C), as a function of the day of the week in the surveyed hummingbird territories (*n* = 21 territories). The area in violin plots shows the data distribution, and the box plots within each violin plot denote the median and interquartile range along with outliers.

Humingbird behavior differed between weekdays and weekends (Fig. 3, Table 2). On weekdays, Broad-tailed hummingbirds made fewer chases against intruders (*β* ± se = 1.66 ± 0.08; *r*^2^ = 86.6%) and visited less flowers within their territories (*β* ± se = 1.04 ± 0.06; *r*^2^ = 88.3%), compared to weekends. The number of intruders successfully accessing the territories was higher on weekdays (*β* ± se = −1.61 ± 0.06; *r*^2^ = 90.3%). Chases were positively associated with the total number of flowers in the territories (*β* ± se = 0.20 ± 0.07). However, the size of the territory (*n* = 21, range: 1,133 ± 5,306 m^2^) had no effect on this behavior. Neither the number of flowers visited nor the number of successful intruders were associated with the size of the territory or the number of flowers (*n* = 21, range: 297 ± 8295 flowers) they contained (Table 2).

**Table 2:** Results of generalized linear mixed models (GLMM) with Poisson error distribution to test for effects of human activity levels (*i.e.*, weekends *vs.* weekdays), territory size and number of flowers (as covariates) on the behavior of the Broad-tailed hummingbird. Data for (a) chases, (b) number of flowers visited and (c) successful intruders were collected from 21 territories on nine surveys through June to August 2016. Significant results are in bold.

	Estimate	se	*z*-value	*Pr* (>|*z*|)
(a) Chases				
(Intercept)	0.81	0.52	1.56	0.116
Human activity levels	1.65	0.80	20.60	**<0.001**
Territory size	−0.06	0.06	−0.91	0.36
Number of flowers	0.09	0.03	2.84	**0.004**
(b) Number of flowers visited				
(Intercept)	2.70	0.30	8.90	**<0.001**
Human activity levels	1.04	0.03	27.03	**<0.001**
Territory size	0.02	0.03	0.66	0.50
Number of flowers	−0.03	−0.03	0.01	0.09
(c) Successful intrusions				
(Intercept)	3.41	0.42	8.08	**<0.001**
Human activity levels	−1.60	0.06	−25.39	**<0.001**
Territory size	−0.01	0.05	−0.19	0.84
Number of flowers	−0.02	0.02	−0.79	0.42

## Discussion

We found that the level of human activities related to agriculture showed a weekly cycle of variation at our study site. On weekdays was an increase in the traffic of pedestrians, cyclists, dogs, farm animals and vehicles compared to the weekends. Our data supported that these differences between weekdays and weekends led hummingbirds to adjust their territorial behavior to these weekly cycles of human activity levels. Compared to weekends, on weekdays hummingbirds showed a decrease in defense (number of chases) as well as the use of their territory (number of flowers visited), which led to increased access by intruders (number of visited flowers by intruders).

Overall, the effect sizes of an increased human activity on hummingbird territorial defense, feeding activity, and intruder access were larger (∼86 to 90%), so their biological relevance is discussed below. Here, we have shown that hummingbirds reduce their territorial defense when human activity levels are higher. At a risky situation (*e.g.*, pedestrian presence), these birds showed great nervousness before moving away from territories and occupying areas close to the shrub cover (presumably to increase protection), suggesting that tolerance may decrease as the stimulus gest closer. Although hummingbirds returned to their perches and defended against intruders once the threat had passed, an increase in the frequency of these events on weekdays makes territories more accessible as owners reduce the number of chases. Thus, although hummingbirds are nimble flyers and probably difficult to capture, they seemed to be aware of the increased human activity in the area, and their behavior suggests that this was incorporated into their decisions while defending a territory. That is, hummingbirds may modify their behavior when they perceive direct human, vehicle, or dog presence as a threat, as seen by the decline in chasing behavior. A variety of different cues (*e.g.*, visual, auditory, olfactory) are used to detect the presence of a risk, such as a predator (Dusenbery, 1992). In birds, many species forage in flocks, which allows more individuals to see a predator and warn of impending danger (Lebar Bajec & Heppner, 2009). However, some bird species, such as hummingbirds, are solitary foragers and cannot rely on conspecifics to warn of impending danger. Therefore, a hummingbird’s rapid assessment of risk in the face of human activity should result in the modification of its behavior to counteract that risk. This dynamic level of human activity has been previously shown to affect territorial behavior in birds from urban environments (*e.g.*, Blumstein et al., 2017; Fernández-Juricic & Tellería, 2000), and there is growing evidence of the way bird species face this challenge. For example, higher human intrusion (Gutzwiller et al., 1994) and/or anthropogenic noise (Zwart et al., 2016), have been shown to promote a reduction in the occurrence and consistency of singing behavior in birds, which suppresses territorial defense. Likewise, anthropogenic activities that increase the density of power lines and human settlements may cause territorial abandonment, as occurs in the critically endangered Bearded Vulture (*Gypaetus barbatus*; Krüger, Simmons & Amar, 2015). Even low levels of recreational human disturbance (*i.e.,* pedestrians) could greatly affect the density and diversity of breeding birds and thereby reduce territory establishment (Bötsch, Tablado & Jenni, 2017). In our study, Broad-tailed hummingbirds chased more intruders on weekends compared to weekdays, when there is more human activity. Thus, the increase in human activity seems to be associated with lower territorial defense effort, but it also suggests that hummingbirds can resume normal behaviors relatively quickly when human disturbance ceases or diminishes (Higham & Shelton, 2011; Titus, Daly & Exton, 2015).

We have shown that during days of increased human activity, Broad-tailed hummingbirds reduce foraging (number of flowers visited) in their territories. Foraging success is a fundamental determinant of animal fitness, and our data suggest that foraging success of hummingbirds under highly disturbed conditions may be less than half that of less disturbed periods (*i.e.,* visiting fewer than 20 flowers on weekdays compared to more than 40 flowers on weekends). At face value, this would likely result in significant fitness costs. However, the reduction in weekday foraging may be largely offset by increased foraging effort during low-risk periods (weekends), since animals should tend to allocate foraging and anti-predator behavior according to temporal variation in risk (Lima & Bednekoff, 1999). In fact, hummingbirds may reduce their energy intake rate in response to predation risk, as previously shown by Lima (1991). Several bird species have demonstrated behavioral choices that favor avoiding perceived risk over acquiring food in an environment with human disturbance. For example, previous studies have shown that the number and activity of people, vehicles and even the presence of free-running dogs, significantly reduced the time that shorebirds and waterbirds spent foraging on beaches or estuarine mudflats (*e.g.*, Burger, 1994; Galicia & Baldassarre, 1997; Burger & Gochfeld, 1998; Thomas, Kvitek & Bretz, 2003; Ramli & Norazlimi, 2017). Similar effects have been documented in various landbird species (*e.g.*, Cooke, 1980; Burger & Gochfeld, 1991; Gutzwiller et al., 1998; Fernández-Juricic, Jimenez & Lucas, 2002). As predicted by the risk-disturbance hypothesis, birds seek a balance between avoiding disturbance and pursuing foraging in response to human presence (Frid & Dill, 2002). Our findings suggest that Broad-tailed hummingbirds can resume foraging activity as usual during low-disturbance periods, which is similar to earlier research showing that birds compensate for the energy losses by increasing food intake following disturbance events (*e.g.*, Tarjuelo et al., 2015). Because hummingbirds maximize their net rate of energy intake by selecting high-reward flowers, even long temporal gaps between foraging bouts can be energetically compensated by these flowers (Tamm & Gass, 1986). At our study site, *Bouvardia ternifolia* flowers provide high nectar rewards (*e.g.*, nectar volume: 4 to 5 µl; sugar production: 0.231 to 0.242 mg mL^−1^; Lara, 2006). Thus, the potential costs of territorial defense and foraging on days of increased human activity may be offset by the food available in their territories. This may explain why no territories were abandoned during the study. Alternatively, hummingbirds facing intense human activity during the weekdays, might forage later in the day, when human activity is low (*e.g.*, resulting in no difference in energy intakes between weekdays and weekends). Our monitoring schedules prevent us from asserting this, so future research should consider morning and evening periods to assess these behaviors.

Hummingbirds frequently act aggressively and territorially by chasing away intruders. The increase in human activity level decreased chasing behavior in our study site, which allowed intruders to use the floral resource within the territory. These results suggest that hummingbirds would be upset by their presence and divert invaders’ focus to vigilance or fly away from these distractions when pedestrians, dogs, farm animals, and vehicles gradually encroach throughout the weekdays. A large number of studies on human-induced behavior change have focused on human impacts on vigilance and fleeing behaviors, which have been used as measures of an animal’s fearfulness and are considered to indicate varying levels of tolerance (Frid & Dill, 2002; Blumstein et al., 2003; Stankowich, 2008; Weston et al., 2012). Changes in both alert activities may also indicate trade-offs with other behaviors such as foraging and territorial defense, particularly because an increasing allocation (*e.g.*, energy or time) to one behavior could requires a decrease in another (*e.g.*, Tarjuelo et al., 2015; Klett-Mingo, Pavón & Gil, 2016). As our data suggest, hummingbirds may be less efficient in territorial defense when there is human disturbance, resulting not only in decreased forage intake within their territories, but an increase of successful access of intruders. However, this allocation of energy and time towards defense behavior may have a net benefit (Pyke, Pulliam & Charnov, 1977; Carpenter et al., 1991). For example, Gill & Wolf (1975) found that having access to an energy-rich resource reduced the amount of time and energy spent foraging, which helps offset the extra energy used for territorial behavior. In a situation where there is dispersed spatial distribution of high-quality resources, being territorial is no longer beneficial as competition will be high which reduces feeding time further whilst increasing energy expenditure for territorial behaviors (Rousseau, Charette & Bélisle, 2014). However, the effect of human disturbance on the territorial behavior of Broad-tailed hummingbirds seems to be buffered by the highly aggregated spatial distribution of *Bouvardia ternifolia* flower patches at our study site, leading owners to remain in their territories even when human presence is at its most intense during weekdays.

While land use change is often considered to have a negative effect on hummingbird populations, particularly in species with greater habitat specialization (Graham & Blake, 2001; Hadley & Betts, 2009; Hadley et al., 2017), it is generally assumed that hummingbirds are tolerant to human disturbance (*e.g.*, Feinsinger et al., 1988; Stouffer & Bierregaard, 1995; Renjifo, 2001). In fact, it has been suggested that the abundance of some species has even been favored in anthropic environments, where the use of feeders and/or the establishment of urban gardens or agrosystems with a wide variety of exotic plants provide permanent sources of food. For example, a subspecies of Allen’s Hummingbird (*Selasphorus sasin sedentarius*) expanded its population in urban Southern California (Clark, 2017). Similarly, Anna’s Hummingbird (*Calypte anna*) and Black-chinned Hummingbird (*Archilochus alexandri*) have expanded their ranges in the United States, presumably due to urbanization and associated increases in nectar plant availability (Zimmerman, 1973; Emlen, 1974). Greig, Wood & Bonter (2017) found that the northward range expansion of wintering Anna’s Hummingbird was significantly related to human-modified habitats and supplemental feeding. This could be the case for Broad-tailed hummingbirds at our study site, as this species is found in different habitat types with varying degrees of human-induced disturbance (Lara, 2006). Hummingbirds are able to fly through wide, disturbed habitats given its high mobility (Hadley & Betts, 2009) and a generalist diet, which makes them more resilient to disturbance than other bird guilds like insectivores (Stouffer & Bierregaard, 1995). Thus, hummingbirds facing weekly cycles of activity at our study site, can easily move to neighboring sites such as the forested areas(particularly when human activity is more intense), where other floral resources are also abundant (*e.g.*, *Penstemon roseus*; Lara, 2006). However, the fact that these hummingbirds are still present in these altered habitats does not necessarily mean that disturbance has no effect on them. These apparent positive effects on taxonomic diversity and/or demographic aspects may be masking a detrimental effect on trait diversity (such as territorial behavior), which appears to be particularly evident at the landscape scale (Flynn et al., 2009; Tinoco, Santillán & Graham, 2018). Many animals are becoming more tolerant of people and acclimating to these altered habitats over time, but some seem to be having effects that we continue to ignore. For example, Samia et al. (2015) examined characteristics of animals that help determine the extent to which they tolerate human presence. In comparison to smaller bird species like hummingbirds, they found that larger bird species like pelicans (genus *Pelecanus*) and black-backed gulls (*Larus marinus*) were more tolerant of humans. This disproves the concept that huge birds are more intolerant of humans and argues that we should pay greater attention to smaller species, especially in light of the fact that tolerance for human presence varies even among tiny species. Such tolerance variation could explain why population trends at large scales reveal dramatic declines for some hummingbird species but increases in others (English et al., 2021), a phenomenon that merits further research.

## Conclusions

Agriculture entails human activity cycles in which working tasks are concentrated on weekdays and reduced on weekends. Larger volumes of people (*i.e.,* pedestrians and cyclists), domestic animals (*i.e.,* dogs and farm animals), and vehicles (*i.e.,* cars and tractors) are present on weekdays compared to weekends. These weekly cycles of human activity influence territorial behavior of the Broad-tailed hummingbirds. Thus, when human activity level is higher (*i.e.,* weekdays), compared to weekends, hummingbirds respond by becoming less aggressive against intruders (*i.e.,* displaying fewer chases), spending less time searching for food (*i.e.,* visiting fewer flowers in their own territory), which leads to an increase in the number of intruders successfully accessing their territories (*i.e.,* visiting flowers). Despite this, our study indicates that hummingbirds may adjust their behaviors during times of minimal disturbance.

##  Supplemental Information

10.7717/peerj.14953/supp-1 Supplemental Information 1 R scripts to analyze raw data (Human activity levels inside the territories on weekends and weekdays) Scripts to obtain GLMM, standardized model coefficients, the effect sizes, and to plot Figure 2.

10.7717/peerj.14953/supp-2 Supplemental Information 2 Raw data showing all hummingbird territories monitored through the study which faced different human activity levels These human activities were used for statistical analysis to compare their frequencies on weekdays and weekends.

10.7717/peerj.14953/supp-3 Supplemental Information 3 R scripts to analyze raw data (testing the effect of weekly cycles of human disturbance on hummingbird territorial behavior) Scripts to obtain GLMM, standardized model coefficients, the effect sizes, and to plot Figure 3.

10.7717/peerj.14953/supp-4 Supplemental Information 4 Raw data showing behaviors recorded in territorial hummingbirds which faced different human activity levels These behaviors were used for statistical analysis to compare their frequencies on weekdays and weekends.

## Additional Information and Declarations

### Data Availability

The following information was supplied regarding data availability: The raw data is available in the Supplemental Files.
